# Spermidine protects from age-related synaptic alterations at hippocampal mossy fiber-CA3 synapses

**DOI:** 10.1038/s41598-019-56133-3

**Published:** 2019-12-23

**Authors:** Marta Maglione, Gaga Kochlamazashvili, Tobias Eisenberg, Bence Rácz, Eva Michael, David Toppe, Alexander Stumpf, Alexander Wirth, André Zeug, Franziska E. Müller, Laura Moreno-Velasquez, Rosanna P. Sammons, Sebastian J. Hofer, Frank Madeo, Tanja Maritzen, Nikolaus Maier, Evgeni Ponimaskin, Dietmar Schmitz, Volker Haucke, Stephan J. Sigrist

**Affiliations:** 1 Department of Biology, Chemistry, Pharmacy, Freie Universität Berlin, 14195 Berlin, Germany; 2 Department of Molecular Pharmacology and Cell Biology, Leibniz Forschungsinstitut für Molekulare Pharmakologie (FMP), 13125 Berlin, Germany; 3 NeuroCure Cluster of Excellence, Charité Universitätsmedizin, 10117 Berlin, Germany; 4 Institute of Molecular Biosciences, NAWI Graz, University of Graz, 8010 Graz, Austria; 5 BioTechMed-Graz, 8010 Graz, Austria; 6 Department of Anatomy and Histology, University of Veterinary Medicine Budapest, 1078 Budapest, Hungary; 7 Charité – Universitätsmedizin Berlin, corporate member of Freie Universität Berlin, Humboldt-Universität zu Berlin, and Berlin Institute of Health, 10117 Berlin, Germany; 8 Cellular Neurophysiology, Hannover Medical School, 30625 Hannover, Germany

**Keywords:** Neural ageing, Long-term potentiation

## Abstract

Aging is associated with functional alterations of synapses thought to contribute to age-dependent memory impairment (AMI). While therapeutic avenues to protect from AMI are largely elusive, supplementation of spermidine, a polyamine normally declining with age, has been shown to restore defective proteostasis and to protect from AMI in *Drosophila*. Here we demonstrate that dietary spermidine protects from age-related synaptic alterations at hippocampal mossy fiber (MF)-CA3 synapses and prevents the aging-induced loss of neuronal mitochondria. Dietary spermidine rescued age-dependent decreases in synaptic vesicle density and largely restored defective presynaptic MF-CA3 long-term potentiation (LTP) at MF-CA3 synapses (MF-CA3) in aged animals. In contrast, spermidine failed to protect CA3-CA1 hippocampal synapses characterized by postsynaptic LTP from age-related changes in function and morphology. Our data demonstrate that dietary spermidine attenuates age-associated deterioration of MF-CA3 synaptic transmission and plasticity. These findings provide a physiological and molecular basis for the future therapeutic usage of spermidine.

## Introduction

Aging is the major risk factor driving age-dependent memory impairment (AMI) even in the absence of neurodegenerative diseases, representing one of the top health burden of the 21^st^ century^1^. Rather than gross neuronal loss characteristic of neurodegenerative diseases^2^, AMI is associated with subtle but selective and region-specific changes such as dysregulation in synapse number and structure, dendrite morphology, cellular connectivity and/or Ca^2+^ homeostasis^3–5^. Aging has also been postulated to be associated with a decline in autophagy/lysosomal proteolysis, a cytoprotective degradation pathway responsible for the turnover of long-lived aggregate-prone proteins and damaged organelles (importantly mitochondria). Autophagy is considered to exert protective functions against aging and age-associated diseases^6^. Irrespective of the exact and potentially complex relation of aging and protective programs such as autophagy, stimulation of autophagy has been proposed to protect from aging effects in various models^7–9^. We have previously demonstrated that feeding fruit flies with the naturally occurring polyamine spermidine, whose levels decline with age in a broad spectrum of organisms likely including humans^7,9–12^, protects from AMI. Spermidine supplementation counteracts ultrastructural and functional changes at aging synapses via a mechanism that requires a functional autophagy machinery^9,11^. First analysis in aging mice has shown that spermidine feeding prolongs life span and exerts cardioprotective effects^7,13^ by autophagic reprogramming^13,14^. Interestingly, a recent pilot trial study indicates that spermidine might exert beneficial effects on cognitive performance in elderly humans at risk of dementia^15^. Thus, taken together, spermidine is a promising candidate for anti-AMI interventions.

Synaptic plasticity in the hippocampus, a pivotal cortical area for learning and memory, is particularly sensitive to aging^16,17^ and age-induced impairment of hippocampus-dependent memories in mice has been suggested to be linked to the downregulation of select autophagy factors^8,18^. Two well-studied types of hippocampal synapses are the (i) mossy fiber (MF)-CA3 synapses formed by dentate granule cell (DG) axons onto CA3 pyramidal neurons and (ii) the CA3-CA1 synapses of the Schaffer collateral pathway. MF-CA3 and CA3-CA1 Schaffer collateral synapses display distinct pre- and postsynaptic forms of long-term potentiation (LTP), respectively^19^, and, hence, might be differentially affected by dietary spermidine in aging animals.

Here we show that both types of hippocampal synapses suffer from an aging-induced decrease in synaptic vesicle (SV) density and mitochondria abundance. Dietary spermidine supplementation protected from age-related synaptic alterations and partially restored defective LTP selectively at hippocampal mossy fiber (MF)-CA3 but not CA3-CA1 synapses while preventing the aging-induced loss of neuronal mitochondria at both types of synapses. Our results unravel synapse-specific beneficial effects of spermidine at hippocampal synapses characterized by differential synaptic plasticity, thereby providing information for the future development of potential therapies against AMI.

## Results

### Dietary spermidine supplementation age-protects autophagy in the mouse hippocampal CA3 area

**Figure 1:**
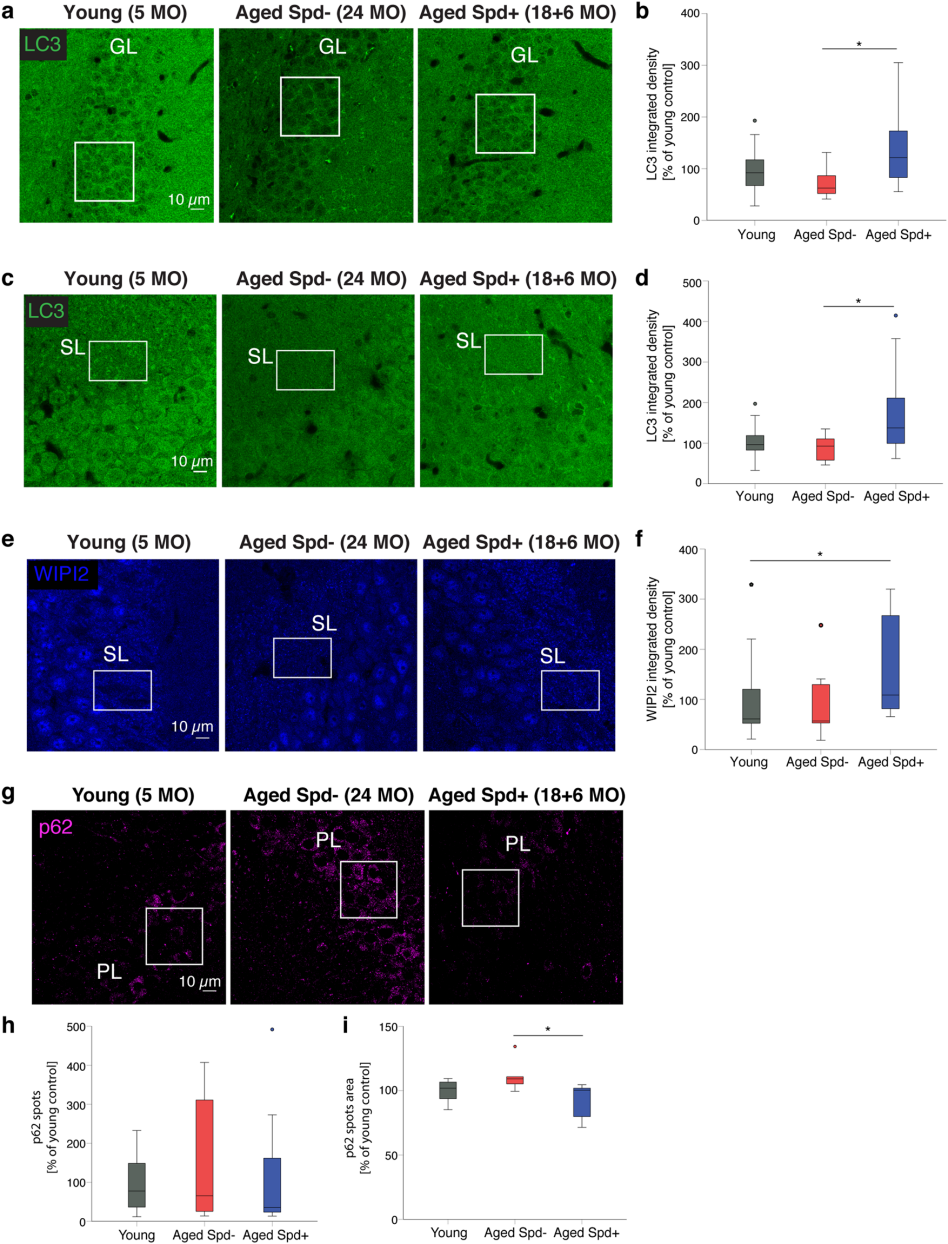
Spermidine treatment increases LC3 and WIPI2 levels while reducing p62 in aged hippocampal neurons. (**a**) Confocal images of LC3 immunoreactivity in the dentate gyrus granule cell layer (GL) of young (5 months old), aged control (24 months old) and aged spermidine treated (18 + 6 months) mice. (**b**) GL LC3 integrated density (young: 100 ± 13, n = 14 mice; aged Spd−: 72 ± 9, n = 10 mice; aged Spd +: 134 ± 21, n = 12 mice, aged Spd− versus aged Spd+: p = 0.026907, one-way ANOVA with Tukey post-hoc test). (**c**) Representative confocal images of LC3 immunoreactivity in the CA3 stratum lucidum (SL) of young, aged control and aged spermidine treated mice. (**d**) SL LC3 integrated density (young: 100 ± 12, n = 14 mice; aged Spd−: 88 ± 9, n = 10 mice; aged Spd+: 172 ± 32, n = 12 mice; aged Spd− versus aged Spd +: p = 0.013770, Kruskal-Wallis test followed by Mann Whitney U test with Bonferroni correction with α set to 0.016667). Results represent 1–2 technical replicates. (**e**) Confocal images of WIPI2 immunoreactivity in the CA3 SL of young, aged control and aged spermidine treated mice. (**f**) SL WIPI2 integrated density (young: 100 ± 24, n = 14 mice; aged Spd−: 88 ± 22, n = 10; aged Spd+: 159 ± 29, n = 12 mice; young versus aged Spd+: p = 0.012670, Kruskal-Wallis test followed by Mann Whitney U test with Bonferroni correction with α set to 0.016667). (**g**) Confocal images of p62 immunoreactivity in the CA3 pyramidal layer (PL) of young, aged control and aged spermidine treated mice. (**h**) PL p62 spots number normalized to young controls (young: 100 ± 24, n = 10 mice; aged Spd−: 148 ± 68, n = 6 mice; aged Spd+:130 ± 69, n = 7 mice). (**i**) PL p62 spots area normalized to young controls (young: 100 ± 3; aged Spd−: 111 ± 5; aged Spd+: 91 ± 5; aged Spd− versus aged Spd+: p = 0.013986, Kruskal-Wallis test followed by Mann Whitney U test with Bonferroni correction with α set to 0.016667). ROIs indicate the area quantified within each image. *p < 0.05. Values represent mean ± SEM. Graphs show medians, interquartile ranges and min/max values. Circles are outliers and pentagons are extremes.

Based on previous studies demonstrating that spermidine supplementation stimulates autophagy^7,20^, we set up several independent cohorts of aged mice undergoing a six-months long spermidine treatment in their drinking water, starting at 18 months of age. To determine if spermidine (Spd) treatment was able to protect from aging-induced decline of autophagy, we analyzed the expression levels of the key autophagy protein LC3 in the hippocampus of young, aged Spd− (24 months old) and aged Spd+ (18 + 6 months) mice. We focused on hippocampal granule cells that form MF synapses onto CA3 pyramidal neurons. In the dentate gyrus granule cell body layer, aging tended to decrease LC3 levels. Importantly, these levels were significantly increased by spermidine treatment (Fig. 1a,b). Similar observations were made in the CA3 stratum lucidum area, where granule cells form MF-CA3 synapses. Akin to the dentate gyrus LC3 levels also tended to decline with age in the synaptic CA3 area, while spermidine treatment led to a significant increase in LC3 expression (Fig. 1c,d). These data suggest that dietary spermidine indeed boosts the steady-state levels of the key autophagy protein LC3 in hippocampal granule cells.

Next, we analyzed the expression levels of WIPI2, an essential autophagy protein whose overexpression restores autophagosome biogenesis in aged dorsal root ganglion neurons^21^. Notably, WIPI2 immunoreactivity appeared particularly pronounced in the stratum lucidum (SL), e.g. a region where MFs are located (Fig. 1e). In the CA3 SL spermidine treatment tended to increase WIPI2 levels compared to identically aged controls (Fig. 1e,f) and WIPI2 levels were significantly upregulated in the spermidine treated cohort in comparison to young control mice (Fig. 1e,f).

Reduced autophagic protein turnover is typically associated with the accumulation of p62, an autophagy receptor for aggregated proteins that itself is a substrate for autophagy^8^. Although the number and size of p62 puncta was highly variable in aged mice, we found that spermidine treatment significantly reduced the p62 spot area as compared to aged matched non-spermidine treated controls (Fig. 1g–i). Collectively, these data suggest that dietary spermidine may indeed promote autophagy in the mouse hippocampal CA3 area *in vivo*.

### Dietary spermidine stabilizes and protects mitochondria at aging hippocampal synapses

**Figure 2:**
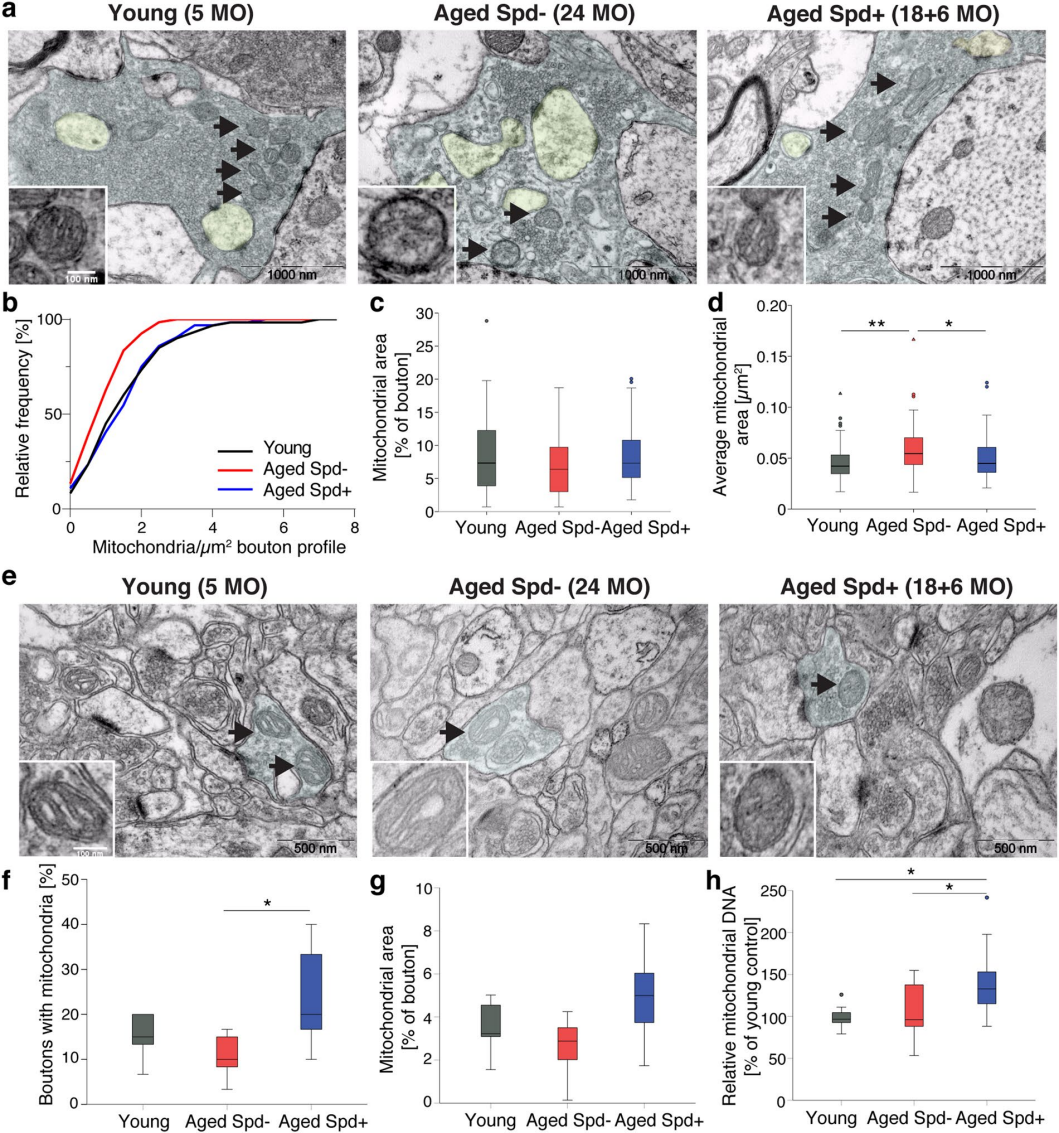
Spermidine protects from age-induced alterations in mitochondria at MF-CA3 synapses by increasing mitochondrial mass. (**a**–**d**) Ultrastructural analysis of Mossy Fiber (MF) terminals. MF bouton area is highlighted in light blue, spiny excrescences in yellow. (**b**) Mitochondria density in MF boutons (p = 0.0046, aged Spd−:1.04 ± 0.09, n = 67 boutons, aged Spd+: 1.65 ± 0.14, n = 64 boutons; p = 0.0142, young: 1.69 ± 0.16, n = 60 boutons, versus aged Spd−, 6 mice/group, two samples Kolmogorov-Smirnov test with Bonferroni correction with α set to 0.016667). (**c**) MF bouton area covered by mitochondria (young: 8.5 ± 0.8, n = 55 boutons; aged Spd−: 6.9 ± 0.6, n = 58 boutons; aged Spd+: 8.4 ± 0.6, n = 56 boutons, 6 mice/group). (**d**) Average visible mitochondrial area (p = 0.011849, aged Spd−: 0.059 ± 0.003, n = 58 boutons, aged Spd+: 0.050 ± 0.003, n = 56 boutons; p = 0.000580, young: 0.046 ± 0.002, n = 55 boutons, versus aged Spd−, 6 mice/group, Kruskal-Wallis test followed by Mann Whitney U test with Bonferroni correction with α set to 0.016667). (**e**–**g**) Ultrastructural analysis of presynaptic CA3-CA1 terminals. (**f**) Percentage of CA3-CA1 presynaptic terminals containing at least one mitochondrion (young: 15 ± 2, n = 6 mice, 180 boutons; aged Spd−: 11 ± 2, n = 7 mice, 210 boutons; aged Spd+: 23 ± 5 n = 6 mice, 180 boutons; aged Spd− versus aged Spd+: p = 0.023280, one-way ANOVA with Tukey post-hoc test). (**g**) Percentage of bouton area covered by mitochondria (young: 3.4 ± 0.5, n = 6 mice, 180 boutons analyzed; aged Spd−: 2.6 ± 0.5, n = 7 mice, 210 boutons; aged Spd+: 4.9 ± 0.9, n = 6 mice, 180 boutons). (**h**) Relative mitochondrial DNA content in brains of young (100 ± 4, n = 9 mice), aged Spd− (106 ± 9, n = 12 mice) and aged Spd + mice (144 ± 14, n = 10 mice), normalized to young controls (young versus aged Spd+: p = 0.021083; aged Spd + versus aged Spd−: p = 0.036320, one-way ANOVA with Tukey post-hoc test). Relative mitochondrial DNA abundance was determined by measuring mitochondrial cytochrome B DNA content relative to nuclear DNA content (β actin). *p < 0.05, **p < 0.01. Values represent mean ± SEM. Graphs show medians, interquartile ranges and min/max values. Circles are outliers and triangles are extremes. Black arrows indicate mitochondria.

Mitophagy, a specialized form of selective autophagy, is an essential mechanism for the long-term maintenance and protection of mitochondrial abundance and functional integrity. Spermidine previously was found to activate the degradation of dysfunctional mitochondria by mitophagy in the heart^7^. We therefore ultrastructurally analyzed mitochondria in presynaptic terminals of MF-CA3 synapses (Fig. 2a–d). Although aging did not alter the percentage of mossy fiber boutons containing at least one mitochondrion (Fig. S1a), the density of mitochondria in aged mossy fiber boutons was significantly decreased compared to MFBs of young adult mice (Fig. 2b), confirming previous observations at mossy fiber boutons of aged rats^22^. Dietary spermidine rescued the age-dependent decrease in mitochondria density at mossy fiber boutons (Fig. 2b), without affecting the fraction of boutons containing mitochondria (Fig. S1a). Interestingly, adult MF terminals often displayed several mitochondria arranged in local networks (Fig. S1b), while in aged mossy fiber boutons such networks tended to decrease (Fig. S1b). Instead, individual mitochondria appeared enlarged, a feature typically associated with a dysfunctional state (Fig. 2a–d). Aging previously was reported to lead to increases in mitochondrial size (“swelling”)^23^, a feature that we also observed in our aged cohort (Fig. 2a,d). Spermidine treatment appeared to protect from this age-induced swelling phenotype and tended to rescue network formation (Figs. 2a,d and S1b). When analyzing the relative bouton area covered by mitochondria in presynaptic MF terminals, we observed a trend towards rejuvenated values in the spermidine supplemented animals (Fig. 2c), though not to statistical significance. To see whether a similar protective effect of spermidine can be observed at other types of hippocampal synapses, we analyzed mitochondrial abundance at CA3-CA1 presynaptic terminals (Fig. 2e–g). At this synapse type, age tended to decrease the number of boutons containing at least one mitochondrion (Fig. 2f). Spermidine treatment significantly increased the fraction of mitochondria-containing boutons (Fig. 2f) and also tended to increase the bouton area covered by mitochondria (Fig. 2g).

Finally, we asked whether the observed increase in presynaptic mitochondrial abundance by spermidine treatment was associated with an overall increase in mitochondrial mass. To probe this, we performed quantitative real time qPCR to measure mitochondrial DNA content of young, aged mock, and aged spermidine-treated brain tissue (Fig. 2h). Analysis of the abundance of mitochondrial DNA (normalized to nuclear β actin DNA) revealed a high variability in aged mice in comparison to young controls. Spermidine treatment significantly increased the relative mitochondrial DNA abundance in comparison to aged mice and young controls (Fig. 2h), indicating that spermidine supplementation can indeed increase net mitochondrial mass.

Taken together, our analyses demonstrate that chronic dietary spermidine supplementation results in age-protection of mitochondria at sites of neurotransmitter release of both MF-CA3 and CA3-CA1 synapses via an overall increase in mitochondrial mass.

### Aging effects on MF-CA3 synapse ultrastructure are responsive to spermidine supplementation

**Figure 3:**
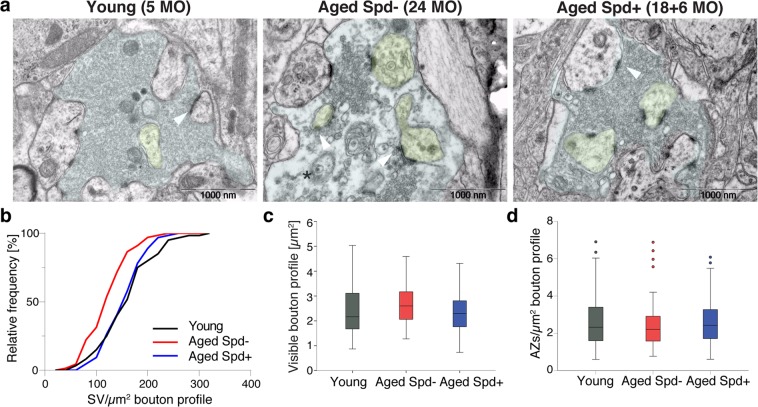
Spermidine treatment rescues the age-dependent decrease in SV density selectively at MF-CA3 synapses. (**a**–**d**) Ultrastructural analysis of Mossy Fiber (MF) terminals. (**a**) Representative electron micrographs of MF boutons. Note the reduction in SV density in an aged Spd− bouton (black asterisk). MF bouton visible area is highlighted in light blue, spiny excrescences are highlighted in yellow. (**b**) Synaptic vesicles (SV) density (p = 0.0004, aged Spd−: 128.26 ± 5.11, n = 67 boutons, versus aged Spd+: 159.43 ± 5.05, n = 64; p = 0.0008, young: 163.03 ± 6.86, n = 60, versus aged Spd−; 6 mice/group; two samples Kolmogorov-Smirnov test Bonferroni corrected with α set to 0.016667). (**c**) Visible bouton profile indicating the synaptic bouton area visible within each image (aged Spd−: 2.69 ± 0.09, aged Spd+: 2.34 ± 0.10, young: 2.43 ± 0.13). (**d**) Active zone (AZ) density (aged Spd−: 2.41 ± 0.15, aged Spd+: 2.62 ± 0.16, young: 2.65 ± 0.19). Arrowheads indicate active zones, note an active zone with drastically reduced neighboring SVs in the representative electron micrograph of an aged control mouse. Values represent mean ± SEM. Graphs show medians, interquartile ranges and min/max values, circles are outliers.

**Figure 4:**
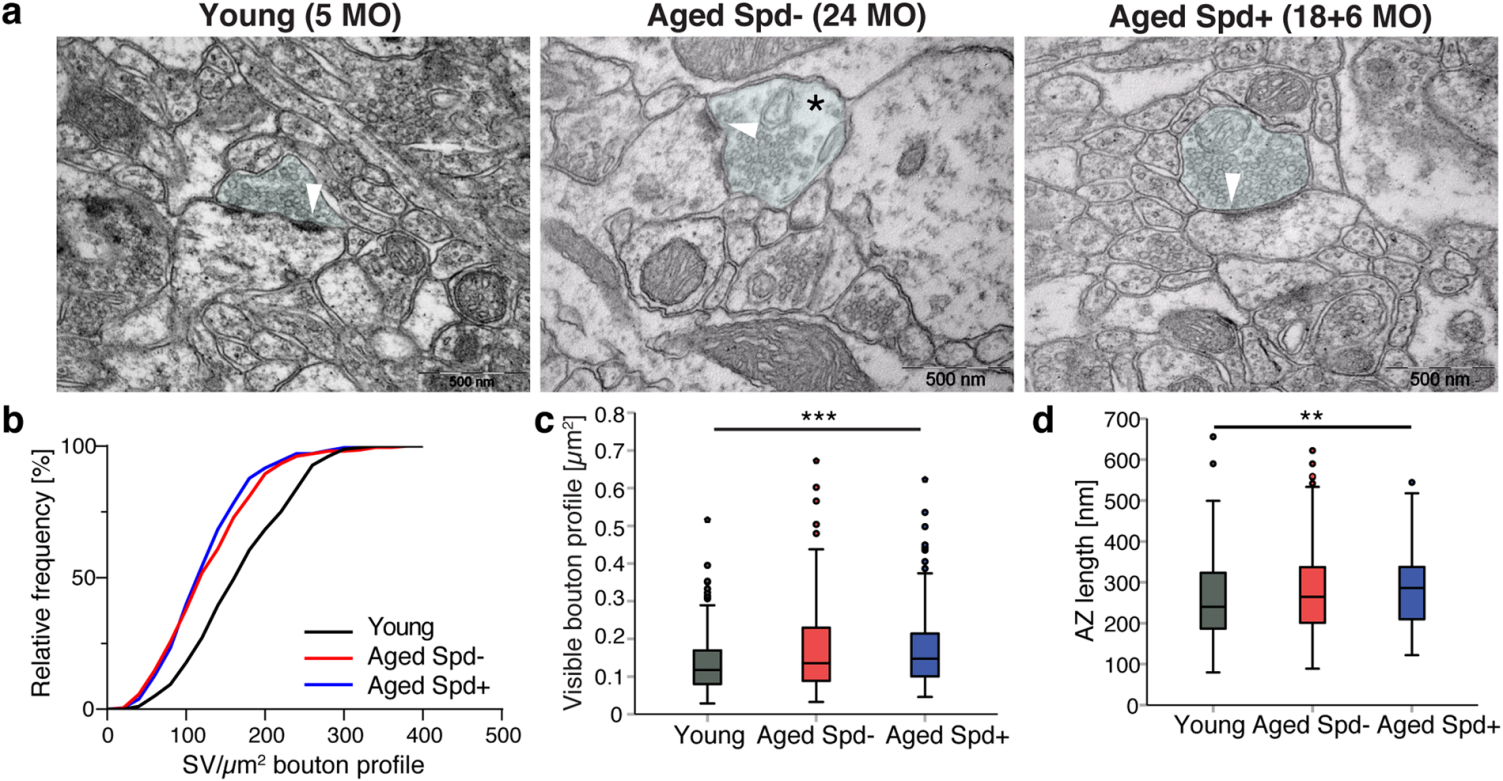
Spermidine treatment does not rescue the age-dependent decrease in SV density at CA3-CA1 synapses. (**a**–**d**) Ultrastructural analysis of presynaptic CA3-CA1 terminals, whose visible area is here highlighted in light blue. (**a**) Representative electron micrographs of CA3-CA1 synapses. Note the reduction in SV density in an aged Spd− bouton (black asterisk). (**b**) Synaptic vesicle (SV) density (young: 175 ± 5, n = 180 boutons, 6 mice analyzed; aged Spd−: 136 ± 4, n = 210, 7 mice; aged Spd+: 130 ± 4, n = 180, 6 mice; young versus aged Spd−: p < 0.0001; young versus aged Spd+: p < 0.0001, two samples Kolmogorov-Smirnov test Bonferroni corrected with α set to 0.016667). (**c**) Visible bouton profile indicating the synaptic bouton area visible within each image (young: 0.135 ± 0.006, aged Spd−: 0.166 ± 0.008, aged Spd+: 0.173 ± 0.008; young versus aged Spd+: p = 0.000106, Kruskal-Wallis test followed by Mann Whitney U test Bonferroni corrected with α set to 0.016667). (**d**) Active zone (AZ) length (young: 260 ± 7, aged Spd−: 280 ± 7, aged Spd+: 287 ± 7; young versus aged Spd+: p = 0.002623, Kruskal-Wallis test followed by Mann Whitney U test Bonferroni corrected with α set to 0.016667). **p < 0.01, ***p < 0.001. Arrowheads indicate active zones, note an active zone with drastically reduced neighboring SVs in the representative electron micrograph of an aged control mouse. Values represent mean ± SEM. Graphs show medians, interquartile ranges and min/max values. Circles are outliers and pentagons are extremes.

Next we asked whether aging promotes changes in ultrastructure at mossy fiber synapses onto CA3 pyramidal neurons (MF-CA3; Fig. 3) and CA3-CA1 synapses (Fig. 4). MF-CA3 synapses display multiple release sites, large paired-pulse and frequency facilitation^19,24,25^ and marked synaptic plasticity of presynaptic origin^19,26^. MF-CA3 synapses thus markedly differ from CA3-CA1 synapses, the vast majority of which form a single synaptic contact^27^. Structural changes at MF-CA3 release sites during aging might therefore conceivably underlie age-dependent alterations in synaptic plasticity at this synapse. EM analysis of the ultrastructure of aged hippocampal MF-CA3 synapses revealed a significant reduction in synaptic vesicle (SV) density by approximately 30% when compared to synapses from young control mice, while the area occupied by mossy fiber boutons remained unchanged (Fig. 3a–c), similar to previous reports for mossy fiber boutons in aged rats^22^. While aging did not significantly alter the density of active zones (Fig. 3d), the length of individual active zones also significantly decreased with age (Fig. S2). Importantly, spermidine treatment for 6 months rescued the age-dependent reduction in SV density of aged MF-CA3 synapses to the density observed in young animals (Fig. 3b). Spermidine did not affect active zone number or active zone length when compared to age-matched controls (Figs. 3d, S2).

To see whether a similar protective effect of spermidine is observed at other types of hippocampal synapses, we analyzed the ultrastructure of CA3-CA1 presynaptic terminals (Fig. 4a–d). This widely investigated synapse executes long-term synaptic plasticity postsynaptically^28^. Consistent with our findings at MF-CA3 synapses, we found that aging decreased SV density at CA3-CA1 synapses (Fig. 4b). In contrast to MF-CA3 synapses treatment with spermidine for 6 months did not rescue these changes (Fig. 4a,b). The visible presynaptic bouton profile tended to increase in aged control and spermidine treatment significantly increased it (Fig. 4c), suggesting that spermidine may not be able to counteract aging-induced alterations in presynaptic bouton size of CA3-CA1 synapses. Similarly, aging tended to be associated with a slightly increased active zone (AZ) length, which could not be reversed by spermidine treatment (Fig. 4d).

Collectively, these data show that dietary spermidine supplementation selectively prevents aging-induced loss of SVs at MF-CA3 but not CA3-CA1 synapses in the mouse hippocampus.

### Dietary spermidine age-protects long-term plasticity specifically at MF-CA3 synapses

**Figure 5:**
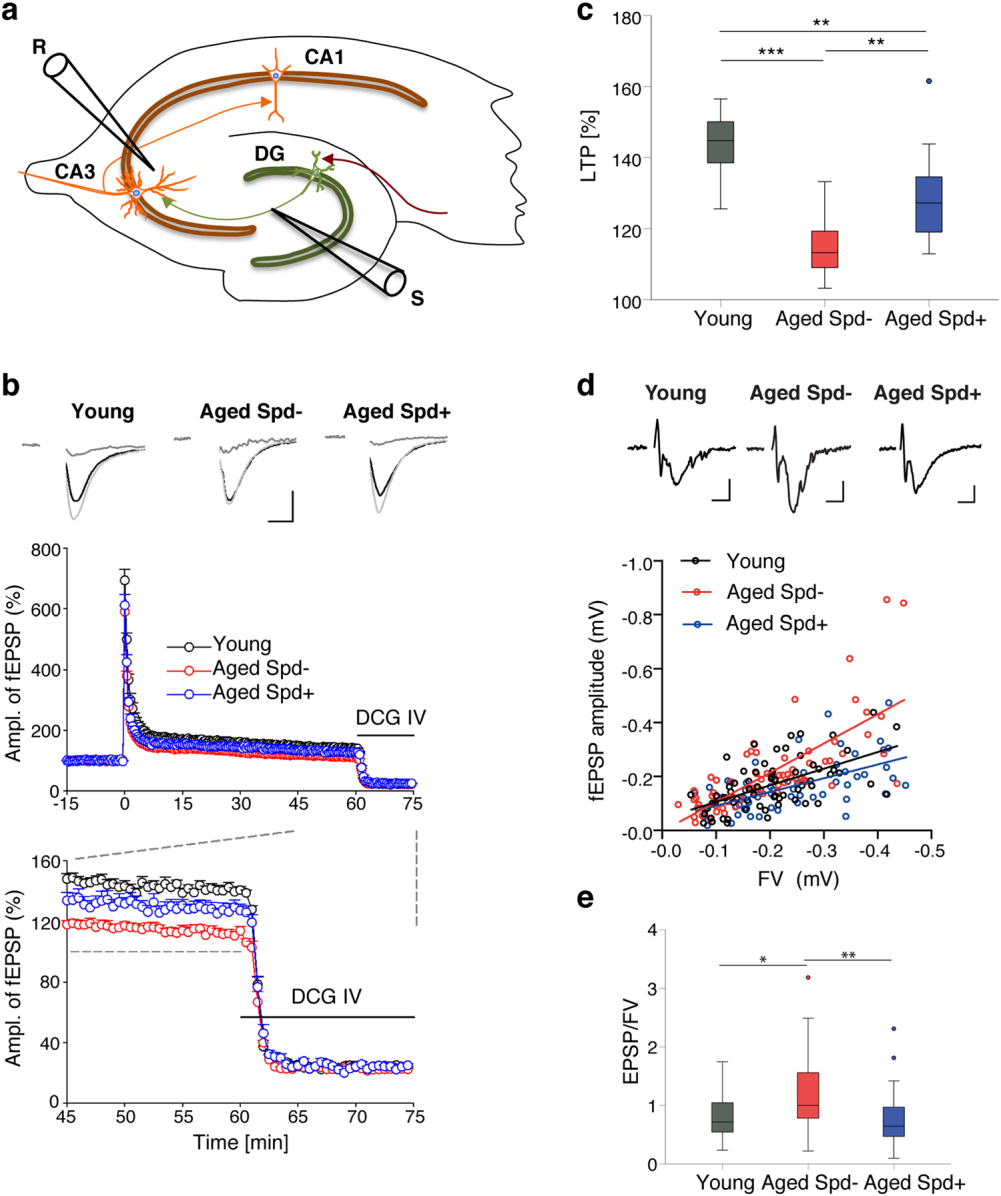
Spermidine treatment rescues the age-dependent decrease in LTP selectively at MF-CA3 synapses while restoring their EPSP/fiber volley curves. (**a**) Scheme of electrophysiological recordings at MF-CA3 synapses, depicting stimulation (S), recording (R) electrode. (**b**) HFS induced LTP of MF-fEPSPs in young (143.1 ± 2.7), aged (114.9 ± 2.5) and spermidine treated mice (130.1 ± 4.5) and averaged MF-fEPSPs collected before (black), 45–60 min after LTP induction (gray) and after application of the agonist of type II metabotropic glutamate receptors DCG IV (2 µM; dark gray). Only responses inhibited by 70–80% and more were assumed to be elicited by mossy fiber synapses. Calibration: 0.3 mV, 5 ms. (**c**) LTP levels 45–60 min after induction. Young: n = 12 slices N = 6 mice, aged Spd−: n = 13 N = 7, aged Spd+: n = 10 N = 6 (p = <0.001, One-way RM ANOVA with Shapiro-Wilk post-hoc test: young versus aged Spd−: p < 0.001, aged Spd− versus aged Spd+: p = 0.002, young versus aged Spd+: p = 0.002). (**d**) Correlation of MF-fEPSP amplitude and FV (fiber volley; ANCOVA: p = 0.0003) and representative average traces of MF-fEPSPs with FVs, scaled to similar FV amplitude. Calibration: 0.2 mV, 5 ms. Several stimulation intensities (0–500 µA) were used to record different FV amplitudes (young: 67 data points; aged Spd−: 68; aged Spd+: 58). (**e**) Average fEPSP/FV values for each slice (young: 0.79 ± 0.08; aged Spd−; 1.19 ± 0.11; aged Spd+: 0.76 ± 0.81; young versus aged Spd−: p = 0.011342; aged Spd− versus aged Spd+: p = 0.000997, Kruskal-Wallis test followed by Mann Whitney U test Bonferroni corrected with α set to 0.016667). Young: n = 22 N = 9; aged Spd−: n = 36 N = 14; aged Spd+: n = 33 N =  = 16. *p < 0.05, **p < 0.01, ***p < 0.001. Values and graphs as in Fig. 3.

**Figure 6:**
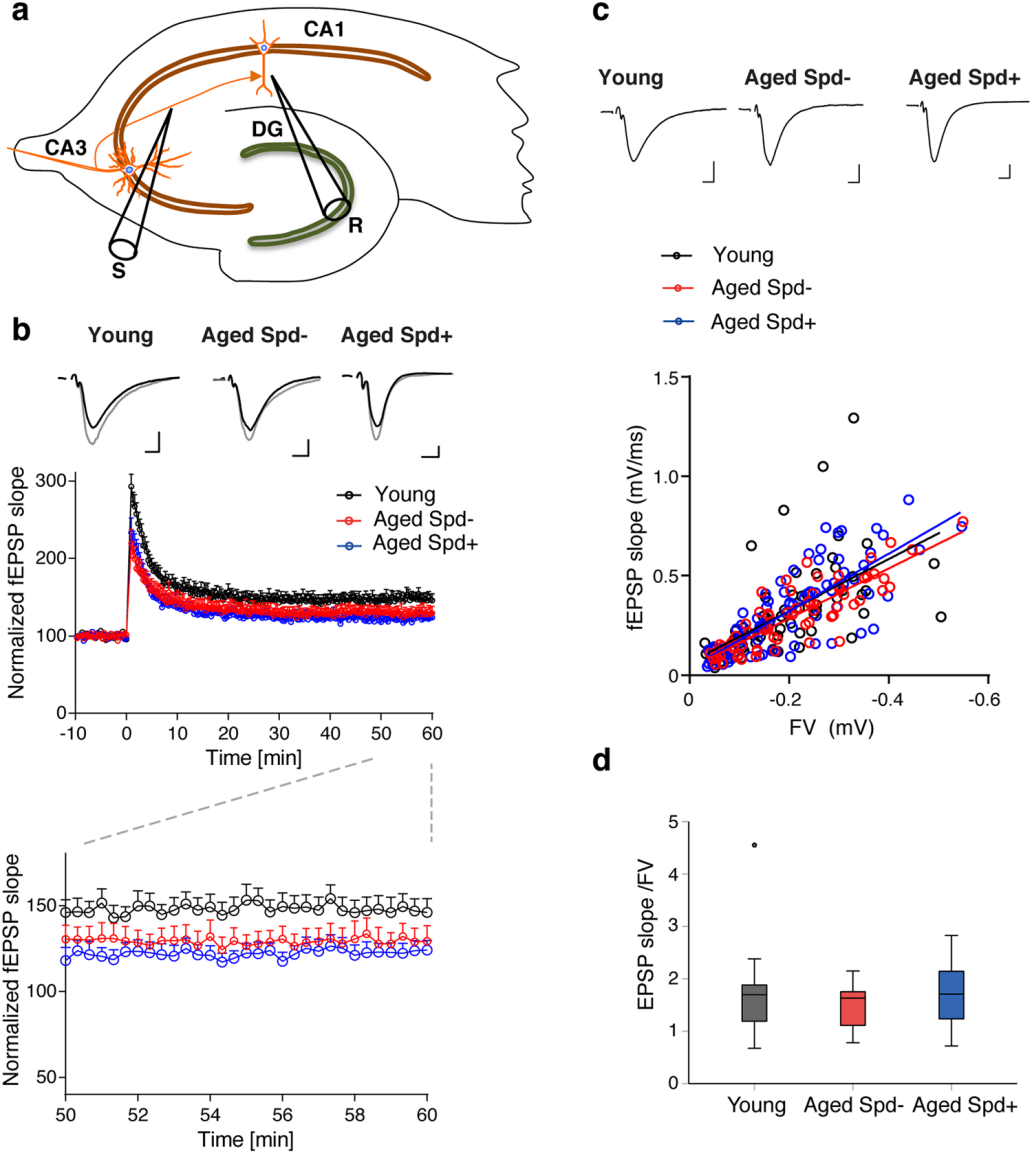
Spermidine treatment does not rescue the age-dependent decrease in LTP at CA3-CA1 synapses. (**a**) Scheme of electrophysiological recordings at hippocampal CA3-CA1 synapses, depicting stimulation (S), recording (R) electrode (Dentate gyrus - DG, CA3 and CA1). (**b**) Time course of LTP induction in CA3-CA1 synapses and average baseline fEPSPs (black) for 10 min before LTP induction and potentiated fEPSPs (gray), 50–60 min after. Young: 145.8 ± 6.9, n = 13 slices N = 5 mice; aged Spd−: 130.6 ± 7.6, n = 10 N = 4; aged Spd+: 123.2 ± 5.4, n = 13 N = 6 (p = 0.0504, One-way ANOVA). Calibration: 0.2 mV, 5 ms. (**c**) Correlation of fEPSP slope and FV (fiber volley) at CA3-CA1 synapses (ANCOVA: p = 0.5754) and average traces of CA3-CA1-fEPSPs with FVs, scaled to similar FV amplitude. Calibration: 0.2 mV, 5 ms. Several stimulation intensities (0–500 µA) were used to record different FV amplitudes; each data point represents the average values for one stimulation intensity (young: 72 data points; aged Spd−: 70 data points; aged Spd+: 85 data points). (**d**) Average CA3-CA1 fEPSP slope/FV values for each slice (young: 1.791 ± 0.26, n = 13 N = 5, aged Spd−: 1.521 ± 0.14, n = 10 N = 4, aged Spd+: 1.707 ± 0.17, n = 14 N = 6; p = 0.7645, Kruskal-Wallis test). Values and graphs as in Fig. 4.

Given these encouraging results, we investigated whether the observed protective effects of spermidine with respect to MF-CA3 synapse ultrastructure would translate into the preservation of synaptic function and plasticity. We therefore analyzed long-term potentiation (LTP), a cellular paradigm for information storage, learning and memory^28^, at MF-CA3 synapses. LTP has been reported to decline with age at hippocampal synapses^29,30^, including MF-CA3 synapses^31^. Indeed, when we analyzed acute hippocampal slices from aged mice (24 months) by field recordings (Fig. 5a), we observed a severe reduction of MF-LTP in comparison to young adult mice (5 months; Fig. 5b,c). Frequency facilitation, a form of short term plasticity pronounced at MF-CA3 synapses in which a switch from low to modest stimulation frequencies lead to an increase in synaptic strength^19^, was unaltered (Fig. S3). Importantly, we found that spermidine supplementation significantly rescued the age-dependent decrease of MF-LTP in 24 months-old animals (Fig. 5b,c). When we analyzed EPSP/fiber volley curves, we observed an age-dependent increase in basal neurotransmission at MF-CA3 synapses that, similar to LTP, was rescued to juvenile levels by spermidine treatment (Fig. 5d,e). As a control we also studied CA3-CA1 synapses that exhibit largely postsynaptic forms of LTP. Consistent with previous studies^29,30^, we observed a trend towards lower LTP of fEPSP slopes at CA3-CA1 synapses of aged animals (Fig. 6a,b). Chronic spermidine administration did not rescue this trend towards an age-dependent decrease in CA3-CA1 LTP (Fig. 6b), consistent with the lack of effect of spermidine with respect to SV density (Fig. 4b). When we analyzed CA3-CA1 EPSP/fiber volley curves, we did not observe any significant changes with either aging or spermidine treatment (Fig. 6c,d). Taken together, our results reveal a synapse-specific restoration of presynaptic LTP at MF-CA3 synapses by dietary spermidine in aged mice.

## Discussion

Elucidating the cellular and molecular mechanisms that underlie the aging of brain function are a major challenge of neuroscience. Finding dietary regimes to fundamentally reduce the rate at which these processes occur is equally relevant. Recent reports suggest that with aging neuronal autophagy might decline^6,8^ and, importantly, that restoring hippocampal autophagy in aged mice allows for a rescue of age-induced memory impairment^8,32^. In the present study, we show that dietary supplementation of spermidine, a substance usually declining with age, can restore levels of core autophagic proteins. We found that spermidine treatment for 6 months starting late in life (at 18 months), a feeding scheme previously shown to extend life-span of aged mice^7^, enhanced clearance of the autophagy substrate p62 and increased expression of key autophagy proteins (LC3, WIPI2). These data are consistent with previous studies in other organ systems demonstrating that spermidine administration can stimulate autophagic flux^20,33–35^ and a prominent autophagic role for WIPI2 in aged neurons^21^. These data thus indicate that chronic spermidine supplementation might promote autophagy in the aged mouse brain.

Age-dependent cognitive decline seems to be tightly associated with subtle synaptic changes but is not mandatorily linked to neuronal cell death^3^, a prominent feature of neurodegenerative disorders rather than normal aging. Changes in the density of the pool of synaptic vesicles distant from active zones (e.g. the recycling and reserve pools) have been previously described at hippocampal Schaffer collateral synapses of aged rats^36^ and at MF-CA3 synapses^22^. Our data confirm that aging indeed leads to decreased SV density at both MF-CA3 and CA3-CA1 synapses (Figs. 3a,b and 4a,b). Most importantly, we found that spermidine treatment late in life could rescue the age-dependent reduction in SV density and in LTP at MF-CA3 synapses. Strikingly, this phenotype was synapse-specific as spermidine administration failed to elicit postsynaptic LTP at and to restore SV density at CA3-CA1 synapses (Figs. 5b,c and 6b). Spermidine supplementation also rescued the age-dependent increase in EPSP/fiber volley amplitudes observed at MF-CA3 synapses (Fig. 5d,e), which might indicate higher basal neurotransmission at these synapses with age, as previously reported for aged synapses in the *Drosophila* olfactory circuitry^11^. Our results are in contrast with a recent study at aged MF-CA3 synapses in rats using 4-6 week old animals as young controls^31^, which might reflect developmental changes still occurring at this early age. The discrepancy might be due to the different species and age windows used.

Different from CA3-CA1 synapses, MF-CA3 synapses are characterized by presynaptic plasticity, a process controlled by presynaptic cAMP and cAMP-dependent protein kinase (PKA) via Rab3a, a SV protein that regulates SV fusion^19,37,38^. Interestingly, we observed similar beneficial effects of spermidine at *Drosophila* mushroom body synapses that are also characterized by cAMP-driven presynaptic plasticity to form new memories^9,11,39^. Our results might indicate that spermidine acts on a mechanism that specifically regulates the SV pool at MF terminals. This is not unlikely considering that MF-CA3 synapses are remarkably different in term of structure, release probability and plasticity from CA3-CA1 synapses^19,24–27,40^. MF-CA3 synapses display multiple release sites, low release probability^41^, strong facilitation and execute long term plasticity via presynaptic mechanisms. In contrast, CA3-CA1 synapses form a single synaptic contact and are characterized by higher release probability^42,43^. Past studies have demonstrated a differential regulation or role of proteins involved in SV cycling at MF-CA3 versus CA3-CA1 synapses, supporting our findings. The Rab3-interacting protein Rabphilin is a PKA effector, that controls the recovery of the ready releasable pool (RRP) of SVs following extensive synaptic activity^44^. Active PKA has been shown to differentially phosphorylate Rabphilin at MF *vs*. CA3-CA1 synapses, suggesting a MF-specific mechanism regulating SVs exocytosis upon RRP depletion^45^. We thus speculate that spermidine might exert beneficial effects specifically at synapses executing synaptic plasticity via presynaptic mechanisms, possibly including autophagic turnover of presynaptic components. While these mechanisms may be complex, our data showing that spermidine supplementation prevents aging-induced defects in presynaptic mitochondria (Fig. 2), suggests a possible role for mitochondrial maintenance in presynaptic Ca^2+^ homeostasis^46–48^. Interestingly, spermidine has been found to increase the rate and affinity of Ca^2+^ uptake in brain mitochondria^49^. This may be of particular importance for pre- but not postsynaptic forms of LTP, i.e. at MF-CA3 synapses. Future studies will be needed to address this possibility. We would like to note that effects of spermidine on CA1 mitochondrial morphological parameters could not be observed in another cohort with very different housing conditions (smaller group size, different environmental enrichment) in a different animal facility which also showed less clear aging effects (data not shown). This suggests that environmental factors can impinge on neuronal ultrastructure of the aging brain per se or on the effects of spermidine in particular. Defining these factors should also be subject of future research.

Taken together, we provide a direct demonstration of the beneficial effects of dietary spermidine supplementation in an electrophysiological paradigm of learning in a mammalian model. Our findings may, thus, be of importance for the development of future therapies against AMI.

## Material and Methods

### Spermidine supplementation

C57BL6 WT mice were purchased from Janvier Labs (C57BL/6 J:Rj males). Spermidine supplementation at a final concentration of 3 mM in drinking water started late in life (18 months of age) for 6 months^7^. A more detail description of housing conditions is found in the Supplemental Information. Notably, the effects of spermidine on CA1 mitochondrial morphological parameters were not observed in another cohort with very different housing conditions (smaller group size, different environmental enrichment) in a different animal facility (data not shown),

All animal experiments were approved by the animal welfare committee of Charité Universitätsmedizin Berlin, Leibniz Institut für Molekulare Pharmakologie (FMP) and the Landesamt für Gesundheit und Soziales Berlin and by the Bundesministerium für Wissenschaft, Forschung und Wirtschaft, BMWFW, Austria: BMWF-66.007/0011-II/3b/2013, BMWFW-66.007/0002-WF/V/3b/2015. All experiments were performed in accordance with the relevant guidelines and regulations.

### Immunohistochemistry

For immunostaining, 30 µm thick coronal hippocampal sections from all groups were processed simultaneously. Following permeabilization, sections were preincubated with 0.125 M PB containing 5% (vol/vol) donkey serum and 0.3% Triton X-100 (TX-100) for 1 h. Sections were incubated with primary antibodies diluted in 0.3% TX-100 at 4 °C for 48 h, then washed and incubated in secondary antibodies for 2 h, RT. A detailed description of the immunohistochemical procedure is listed in the Supplemental Information.

### Electron microscopy

Following transcardial perfusion with a mixture of 2% formaldehyde (FA) and 2% glutaraldehyde in 0.1 M phosphate buffer (PB, pH 7.4) under anesthesia, brains were post-fixed overnight in 4% FA at 4 °C. Ultrathin sections were examined with a JEM-1011 transmission electron microscope (JEOL, Tokyo, Japan). Data collection was performed blindly. A more detail description is found in the Supplemental Information.

### Ultrastructural analysis

We analyzed six-seven mice per group. Visible bouton area, synaptic vesicles, active zones, mitochondria and their visible area were manually annotated in ImageJ (1.48 v). A more detail description is found in the Supplemental Information.

### Electrophysiology

For recordings of MF-fEPSPs and CA3-CA1 fEPSP, mice were anesthetized with isoflurane overdose and transcardially perfused with ice cold dissection artificial cerebrospinal fluid (ACSF)^50^ or directly decapitated, respectively. 350 μm-thick (MF recordings) or 300 µm-thick (CA3-CA1 recordings) hippocampal sections were incubated at 35 °C for 30 minutes immediately after preparation and kept in a resting chamber at 22–24 °C (MF recordings) or at RT (CA3-CA1 recordings) for at least an hour before use. A detailed description of all the electrophysiological experiments is found in the Supplemental Information.

### Statistic

Statistic was performed with IBM SPSS Statistics version 25 (IBM) or with GraphPad Prism (GraphPad). Data distribution was assessed by Kolmogorov-Smirnov normality test and by inspecting histograms and normality Q-Q plots. Data were analyzed with one-way ANOVA followed by Tukey post-hoc test or by Kruskal-Wallis test followed by Mann Whitney U test, Bonferroni corrected with α set to 0.016667 to account for multiple comparisons, unless otherwise stated. Only two tail p-values were considered. Values are expressed as mean ± SEM, n indicates the number of boutons analyzed for EM analysis or the number of brain slices for electrophysiological experiments, unless otherwise stated. A detailed description of the statistical analysis is found in the Supplemental Information.

## Supplementary information

Maglione_et_al_SI_SciRep_revision

## Data Availability

The data that supports the findings of this study are available from the corresponding authors (VH and SJS) upon reasonable request.
