# Supplementary material for: Spermidine protects from age-related synaptic alterations at hippocampal mossy fiber-CA3 synapses
Source: Sci Rep. 2019 Dec 23;9:19616. doi: 10.1038/s41598-019-56133-3 (PMC6927957; doi:10.1038/s41598-019-56133-3)
Supplement: Supplementary file 1 — Maglione_et_al_SI_SciRep_revision [file 41598_2019_56133_MOESM1_ESM.pdf]

Supplementary Information

Spermidine protects from age-related synaptic alterations at hippocampal mossy fiber-CA3 synapses

Marta Maglione[1,2,3], Gaga Kochlamazashvili[2], Tobias Eisenberg[4,5], Bence Rácz[6], Eva Michael[1,3], David Toppe[1,3], Alexander Stumpf[3,7], Alexander Wirth[8], André Zeug[8], Franziska E Müller[8], Laura Moreno-Velasquez[3,7], Rosanna P Sammons[3,7], Sebastian J Hofer[4,5], Frank Madeo[4,5], Tanja Maritzen[2], Nikolaus Maier[3,7], Evgeni Ponimaskin[8], Dietmar Schmitz[3,7], Volker Haucke[1,2,3,*], Stephan J Sigrist[1,3,*]

[1]Department of Biology, Chemistry, Pharmacy, Freie Universität Berlin, 14195Berlin, Germany
[2]Department of Molecular Pharmacology and Cell Biology, Leibniz Forschungsinstitut für Molekulare Pharmakologie (FMP), 13125Berlin, Germany
[3]NeuroCure Cluster of Excellence, Charité Universitätsmedizin, 10117 Berlin, Germany
[4]Institute of Molecular Biosciences, NAWI Graz, University of Graz, 8010 Graz, Austria
[5]BioTechMed-Graz, 8010 Graz, Austria
[6]Department of Anatomy and Histology, University of Veterinary Medicine Budapest, 1078 Budapest, Hungary
[7]Charité – Universitätsmedizin Berlin, corporate member of Freie Universität Berlin, Humboldt-Universität zu Berlin, and Berlin Institute of Health,10117Berlin, Germany
[8]Cellular Neurophysiology, Hannover Medical School, 30625 Hannover, Germany

[*]To whom correspondence may be addressed. Email addresses: Haucke@fmp-berlin.de, stephan.sigrist@fu-berlin.de

Material and methods

Spermidine supplementation in mice

C57BL6 WT mice were purchased from Janvier Labs (C57BL/6J:Rj males). Spermidine supplementation started late in life (18 months of age) for 6 months as described [1]. Aged animals were randomly divided in two groups: control mice received normal drinking water autoclaved without spermidine, while the second group obtained regular autoclaved drinking water supplemented with spermidine (aqueous stock solution, sterile filtered, pH 7.2) at final concentration of 3 mM. Mice were euthanized for experiments at the age of 24 months. Five months old C57BL/6J:Rj male mice were used as young controls.
Mice were housed in a 12-h light and 12-h dark cycle with access to food (maintenance food, Ssniff, cat. V1534) and water *ad libitum*. Nest material and paper houses were used as cage enrichment. Animals used for electron microscopy analyses were housed in groups of up to 10 animals/cage, under SPF conditions in individually ventilated cages (IVCs Type IIL).
Ultrastructural and electrophysiological analysis were performed on independent cohorts of animals. Immunostaining, electrophysiology and RT-qPCR experiments were performed on at least two mouse cohorts. Data acquisition and analysis was carried out blind to the experimenter (electrophysiology, immunostainings, RT-qPCR and CA3-CA1 EM).
All animal experiments were performed in accordance to directive 2010/63/EU and approved by the animal welfare committee of Charité Universitätsmedizin Berlin, Leibniz-Forschungsnstitut für Molekulare Pharmakologie (FMP), the Landesamt für Gesundheit und Soziales Berlin and by the Bundesministerium für Wissenschaft, Forschung und Wirtschaft, BMWFW, Austria: BMWF-66.007/0011-II/3b/2013, BMWFW-66.007/0002-WF/V/3b/2015.

Immunohistochemistry on brain sections and image analysis

Young (5 months), aged Spd- (24 months) and aged Spd+ (18 + 6 months) were euthanized by an overdose (i.p.) of Ketamin (120 mg/kg body weight)/Xylazin (16 mg/kg body weight) and transcardially perfused with 1x PBS followed by 4% (wt/vol) PFA (Merck) in 0.1 M PBS, pH 7.4. Brains were carefully removed from the skull, postfixed overnight in the same fixative, and placed in a mixture of 20% (vol/vol) glycerol and 2% (vol/vol) dimethyl sulfoxide (DMSO; VWR International) in 0.4 M PB, pH 7.4 for at least 24 h for cryoprotection. Frozen coronal sections (30 µm thick) were collected in six series in the above mentioned DMSO solution. For immunostaining, corresponding hippocampal sections from all groups were processed simultaneously. Sections were washed three times in 0.125 M PB, pH 7.4 (3 × 15 min each), followed by washing several times in 0.125 M PB containing 0.3% Triton X-100 (9 × 20 min each). Sections were pre-incubated with 0.125 M PB containing 5% (vol/vol) donkey serum or normal goat serum and 0.3% Triton X-100 (TX-100) for 1 h, rinsed 3 times and subsequently incubated 1 h RT with Donkey or Goat anti mouse IgG Fab fragment 1:25 in 0.125 M PB to remove unspecific background. After rinsing 3 times with 0.3% TX-100 sections were incubated with primary antibodies diluted in 0.3% TX-100 at 4 °C for 48 h with Mouse anti LC3 (MBL,M152-3) 1:200, Rabbit anti WIPI2 (Sigma, HPA019852-100UL) 1:200 or Guinea Pig anti p62 (Progen GP-62-C). Then, sections were washed nine times for 20 min each in 0.3% TX-100 in 0.125 M PB and incubated with Donkey anti mouse-AF488 (1:400; Jackson Immunoresearch, 715-545-151), Donkey anti rabbit AF647 (1:400, Jackson Immunoresearch, 711-605-152) or Goat anti Guinea Pig AF594 (1:400, Thermo Fisher Scientific, A-11076) for 2 h, RT. To quench lipofuscine autofluorescence, sections were briefly rinsed with distilled water and incubated with 10 mM CuSO4 in 50 mM Ammonium Acetate (pH 5), 1 h at RT, rinsed with distilled water and finally washed 8 times for 5 minutes with 0.125 M PB. Finally, sections were mounted on gelatin-coated glass slides with Möwiol, pH 8 (Roth, 0713) on high precision coverslips (Roth, 1.5H).

Images were acquired with a Leica TCS SP8 confocal microscope (Leica Microsystems) equipped with a 63x oil immersion objective, numerical aperture 1.4. The acquisition was performed with 1024 x 1024 pixels per image and a scanning speed of 400 Hz in a sequential manner, with zoom factor 1.3 and lateral pixel size of 0.139 µm x 0.139 µm. All acquisition settings were set equally for sections of all groups within each immunostaining. Images were quantified with the Fiji/ImageJ software (NIH). Images were thresholded and subsequently quantified by measuring the integrated density in a defined region of interest (ROI) in the acquired hippocampal images. The ROI size was set to 42 µm x 42 µm and to 54 µm x 33µm for granule cell layer and CA3 stratum lucidum, respectively. To quantify p62 spots size and number confocal images were thresholded and particles analyzed with the analyze function within a ROI; only particle with size larger than 2 pixels were selected for analysis. Within one set of experiments, the threshold was kept constant for all images of all groups within one confocal channel. Experiments were performed two-three times on different biological replicates from two to three different mouse cohorts. LC3 data represent 1-2 technical replicates, data from the same replicate were averaged. Data were normalized to the mean of young controls and data collected from different experiments were then pooled together. Experiments and analysis were performed blindly.

Tissue preparation and transmission electron microscopy

Mice were deeply anesthetized with a mixture of Ketamin (100 mg/kg body weight)/Xylazin (5 mg/kg body weight) of body weight and were perfused transcardially with 0.9% NaCl for 1-2 min followed by 200 ml of a mixture of 2% formaldehyde (prepared freshly from

paraformaldehyde by heating at 60 °C) and 2% glutaraldehyde in 0.1 M phosphate buffer (PB, pH 7.4) at a flow rate of 10-15 ml/min. Brains were removed and post-fixed overnight in 4% formaldehyde in PB at 4ºC. 70 micrometer thick sections were cut with a Leica VT1000 vibratome (Wetzlar, Germany). There was no obvious gross evidence of differences in brain size across diets, age, and experimental groups. Vibratome sections for electron microscopy were postfixed with 1% OsO4, dehydrated in ascending ethanol series and embedded in epoxy resin (Durcupan; Sigma, Germany) within Aclar sheets (EMS, Hatfield, PA, USA). Uniform rectangular samples were cut from the dorsal CA3 hippocampus stratum lucidum and from the CA1 stratum radiatum (approx. −4.0 from Bregma) under a Leica S6D dissecting microscope and mounted on plastic blocks. ~60 nm ultrathin sections were cut on a Reichert ultramicrotome, mounted on 300 mesh copper grids, contrasted with lead citrate (Ultrostain II, Leica, Germany) and examined with a JEM-1011 transmission electron microscope (JEOL, Tokyo, Japan) equipped with a Mega-View-III digital camera and a Soft Imaging System (SIS, Münster, Germany) for the acquisition of the electron micrographs. Sample areas (at least 50 μm^2 per animal) were chosen in a pseudo-random fashion and photographed at a uniform magnification. Mossy-fiber (MF) boutons from stratum lucidum of the CA3 area, Schaffer collateral axo-spinous boutons from CA1 stratum radiatum and profiles of mitochondria were identified on electron micrographs. Data collection was performed blindly.

Ultrastructural analysis

For MF-CA3 and CA3-CA1 synaptic terminals, we analyzed six mice and six-seven mice per group, respectively. Only CA3 MF bouton profiles contacting at least either two spiny excrescences or a spiny excrescence and a dendrite, and containing multiple active zones, were considered for analysis. Only CA3-CA1 boutons with a clearly visible bouton membrane and active zone apposed to a post-synaptic density were used for analysis. Visible bouton area, synaptic vesicles, active zones, mitochondria and visible area were manually annotated in ImageJ (1.48v). Synaptic vesicles and mitochondria were counted with the Cell Counter Plugin in ImageJ (1.48v). To account for differences in bouton size, the number of synaptic vesicles and mitochondria was normalized to the visible terminal area for each bouton. Visible mitochondria area was manually traced and the percentage of visible bouton area covered by mitochondria calculated for MF terminals containing at least one mitochondria. MF mitochondria networks were defined as at least three mitochondria in contact with each other within a bouton. Active zones were traced manually and their length annotated. At MF synapses, puncta adherentia, membrane specializations found apposed to dendritic shaft, were recognized by their lack in synaptic vesicles and in widening of the cleft [2] and were excluded from AZ quantification. CA3-CA1 data analysis was performed blindly.

Electrophysiology

Slice preparation: For MF-CA3 LTP and frequency facilitation (FF) recordings, mice were anesthetized with isoflurane overdose and transcardially perfused with ice cold dissection artificial cerebrospinal fluid (ACSF) containing the following substances (in mM): sucrose 75, glucose 25, NaCl 87, NaHCO3 25, KCl 2.5, NaH2PO4 1.25, CaCl2 0.5, MgCl2 7, pH 7.35-7.4 [3]. Dissection ACSF was cooled down in a freezer and bubbled at least 30 min prior to use with 95% O2 / 5% CO2. After 2 minutes of perfusion brain were quickly removed and fresh 350 µm-thick hippocampal sections were prepared from both hemispheres using a vibratome (VT 1200S from Leica, Nussloch, Germany) and kept in sucrose based cutting/storage solution for

recovery[3]. Slices were incubated at 35°C for 30 minutes immediately after preparation and kept in a resting chamber at 22-24°C for at least an hour before use.

For MF-fEPSP/FV comparison and for CA3-CA1 LTP recordings mice were anesthetized with Isoflurane and decapitated. The brain was quickly removed and chilled in ice-cold sucrose-artificial cerebrospinal fluid (sACSF) containing (in mM): NaCl 50, NaHCO3 25, glucose 10, sucrose 150, KCl 2.5, NaH2PO4 1, CaCl2 0.5 and MgCl2 7, continuously oxygenated. Sagittal slices (300 μm) were cut and stored submerged in sACSF for 30 min at 35 °C and subsequently stored in ACSF containing (in mM): NaCl 119, NaHCO3 26, glucose 10, KCl 2.5, NaH2PO4 1, CaCl2 2.5 and MgCl2 1.3 saturated with 95% (vol/vol) O2/5% (vol/vol) CO2, pH 7.4, at room temperature. Experiments were started 1 to 6 h after the preparation.

Recordings of MF-fEPSPs: Recordings were performed in a submerged recording chamber (Warner instruments RC-27L), filled with recording ACSF with solution exchange speed set to 3-5 ml/min at room temperature (22-24°C). Recording ACSF of following composition (in mM): NaCl 120, KCl 2.5, NaH2PO4 1.25, NaHCO3 25, MgS04 1.5, CaCl2 2.5, glucose 25, pH 7.35-7.4 was continuously oxygenated with the same gas mixture as above. MF-fEPSPs were generated using glass stimulating electrode (Hilgenberg) filled with ACSF (1-1.5 MΩ), which was placed near to the internal side of granule cell layer of the dentate gyrus and recording electrode (1.5-2.5 MΩ) in the str. lucidum of the CA3 field. MF responses were identified using frequency facilitation parameter in which stimulation frequency is set to 0.3 Hz in order to detect facilitation of responses (Fig. S3). The responses which exhibit at least 200% facilitation were accepted as MF-fEPSPs and were recorded further. The stimulation intensity for FF and LTP experiments were selected to 50-60 % and four HFS delivered every 30 seconds each one containing 100 pulses at 100Hz were applied to induce LTP. LTP at these synapses can be generated presynaptically and is known to be NMDA receptor-independent, therefore 50 μM APV was bath applied during recordings. In order to confirm that fEPSPs were generated by the stimulation of MFs an agonist of type II metabotropic glutamate receptors, DCG IV (2 μM), was applied and only responses inhibited by 70-80% and more were assumed to be elicited by mossy fiber synapses. Recordings and data quantification was performed blindly.

Input output curves for MF: Recordings were performed in a submerged recording chamber (Warner instruments RC-27L), filled with recording ACSF with solution exchange speed set to 3-5 ml/min at room temperature (22-24°C). Recording ACSF of following composition (in mM): NaCl 119, NaHCO3 26, glucose 10, KCl 2.5, NaH2PO4 1, CaCl2 2.5 and MgCl2 1.3 saturated with 95% (vol/vol) O2/5% (vol/vol) CO2, pH 7.4, at room temperature. Stimulation intensities (0 – 500 μA) were adjusted in order to record different FV amplitudes (0.1 mV increment) in one slice. The amplitudes of fEPSPs were correlated with FV amplitudes and linear regression was performed to calculate the slope. Recordings and data quantification was performed blindly.

Recordings of CA3-CA1-fEPSPs: Recordings were performed in a submerged recording chamber (Warner instruments RC-27L), filled with recording ACSF with solution exchange speed set to 3-5 ml/min at room temperature (22-24°C). Recording ACSF of following composition (in mM): NaCl 119, NaHCO3 26, glucose 10, KCl 2.5, NaH2PO4 1, CaCl2 2.5 and MgCl2 1.3 saturated with 95% (vol/vol) O2/5% (vol/vol) CO2, pH 7.4, at room temperature. Stimulation electrodes were placed in str. radiatum near CA3 to stimulate Schaffer Collaterals and recording electrode in the str. radiatum of the CA1 field. Basal stimulation was applied every 10 sec in order to monitor stability of the responses at least for 10 minutes before LTP was induced by four HFS trains (100 pulses, 100 Hz, 10 s inter train interval). Magnitude of LTP was determined by normalizing the initial fEPSP slope 55 -60 min after LTP induction to average baseline fEPSP slope. Data collection and quantification was performed blindly.

Input output curves for CA3-CA1: Recordings were performed a submerged recording chamber (Warner instruments RC-27L), filled with recording ACSF with solution exchange speed set to 3-5 ml/min at room temperature (22-24°C). Recording ACSF of following composition (in mM): NaCl 119, NaHCO3 26, glucose 10, KCl 2.5, NaH2PO4 1, CaCl2 2.5 and MgCl2 1.3 saturated with 95% (vol/vol) O2/5% (vol/vol) CO2, pH 7.4, at room temperature. Stimulation intensities (0 – 500 µA) were adjusted in order to record different FV amplitudes (0.05 mV increments) in one slice. The initial fEPSP slopes were correlated with FV amplitudes and linear regression was performed to calculate the slope. Recordings and data quantification were performed blindly.

Quantitative real-time PCR

To estimate mitochondrial DNA abundance relative to nuclear DNA content quantitative real-time PCR was performed. First total DNA was isolated from brain parts left over from the electrophysiological preparation, therefore void of the hippocampus, using the spin-column-based isolation NucleoSpin® Tissue kit (Macherey-Nagel, 740952.50), according to manufacturer instructions. The concentration and purity of the DNA for each brain sample was determined with a Nanodrop spectrophotometer (Thermo Scientific). DNA concentration was adjusted to 10 ng/µl. The primers used to amplify mitochondrial *cytochrome B* were *forward*: 5'-CCACTTCATCTTACCATTTATTATCGC-3', *reverse*:5'-TTTTATCTGCATCTGAGTTTAATCCTGT-3'. The primers used to amplify nuclear *β actin* were *forward*: 5-CTGCCTGACGGCCAGG-3', *reverse*:5'-GGAAAAGAGCCTCAGGGCAT-3', as reported in Niemann et al.(2014)[4]. The primers were used at a concentration of 450 nM and each RT-qPCR reaction was carried with 15 ng DNA/sample. RT-qPCR was performed using the Dynamo Flash SYBR green master mix (Thermo-Fischer, F415L) and the Agilent Technologies Stratagene Mx3005P Real-time PCR system, according to the manufacturer's instructions. The C_T-values of triplicate reactions/sample were averaged and the ΔC_T value was calculated by subtracting the mitochondrial DNA C_T-value from the nuclear DNA C_T-value. Relative mitochondrial DNA content was then calculated according to Rooney et al. (2015) using the formula 2 x$2^{\Delta C_T}$[5]. The experiment was performed 3 times on different biological replicates obtained from two different mouse cohorts. Values for all groups were then normalized to the mean of young controls and data collected from different experiments were then pooled together. Experiments and quantification were performed blindly.

Statistical analysis

Statistical analysis was performed with IBM SPSS Statistics version 25 (IBM) or with GraphPad Prism (GraphPad). MF-LTP and FF data were analyzed with SigmaPlot (Systat GmbH) software. Sample sizes for electrophysiological recordings and immunostainings were determined by power analysis based on pilot studies. Sample size for EM analysis was based on previous studies [6,7]. Data distribution was assessed by Kolmogorov-Smirnov normality test and by inspecting histograms and normality Q-Q plots. Data acquisition and analysis was carried out blind to the experimenter (electrophysiology, immunostainings, RT-qPCR and CA3-CA1 EM). Data were analyzed with one-way ANOVA followed by Tukey post-hoc test or by Kruskal-Wallis test followed by Mann Whitney U test, Bonferroni corrected with α set to 0.016667, unless otherwise stated. Prior performing one-way ANOVA, equality of variances was tested with the Levene test. Analysis of Covariance (ANCOVA) was used to test whether slopes of regression lines were significantly different. Values are expressed as mean ± SEM, n indicates the number of boutons analyzed for EM analysis or the number of brain slices for electrophysiological experiments, unless otherwise stated.

References

1 Eisenberg, T. *et al.* Cardioprotection and lifespan extension by the natural polyamine spermidine. *Nat Med* **22**, 1428-1438, doi:10.1038/nm.4222 (2016).

2 Rollenhagen, A. *et al.* Structural determinants of transmission at large hippocampal mossy fiber Synapses. *Journal of Neuroscience* **27**, 10434-10444, doi:10.1523/Jneurosci.1946-07.2007 (2007).

3 Bischofberger, J., Engel, D., Li, L., Geiger, J. R. & Jonas, P. Patch-clamp recording from mossy fiber terminals in hippocampal slices. *Nat Protoc* **1**, 2075-2081, doi:10.1038/nprot.2006.312 (2006).

4 Niemann, A. *et al.* The Gdap1 knockout mouse mechanistically links redox control to Charcot-Marie-Tooth disease. *Brain* **137**, 668-682, doi:10.1093/brain/awt371 (2014).

5 Rooney, J. P. *et al.* PCR based determination of mitochondrial DNA copy number in multiple species. *Methods Mol Biol* **1241**, 23-38, doi:10.1007/978-1-4939-1875-1_3 (2015).

6 Kononenko, N. L. *et al.* Compromised fidelity of endocytic synaptic vesicle protein sorting in the absence of stonin 2. *Proceedings of the National Academy of Sciences of the United States of America* **110**, E526-535, doi:10.1073/pnas.1218432110 (2013).

7 Kaempf, N. *et al.* Overlapping functions of stonin 2 and SV2 in sorting of the calcium sensor synaptotagmin 1 to synaptic vesicles. *Proceedings of the National Academy of Sciences of the United States of America* **112**, 7297-7302, doi:10.1073/pnas.1501627112 (2015).

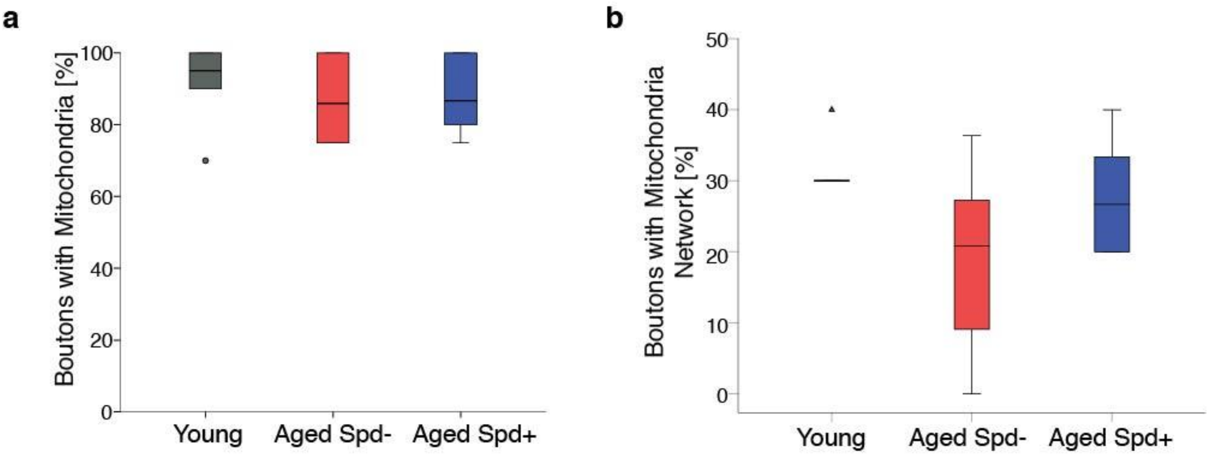

Fig. S1. Spermidine feeding tends to restore mitochondria network without altering the fraction of MF terminals containing at least one mitochondrion. (a) Ultrastructural analysis of the fraction of MF boutons containing at least one mitochondrion in young (5 months), aged controls (Spd-, 24 months) and aged spermidine (Spd+) treated mice (18 months + 6 months treatment; p=0.739256, young: 91.67 ± 4.77, n=6 mice, 60 boutons, aged Spd-: 86.97 ± 4.70, n=6 mice, 67 boutons, aged Spd+: 88.06 ± 4.27, n=6 mice, 64 boutons, Kruskal-Wallis test). (b) Ultrastructural analysis of the fraction of MF boutons containing a mitochondria network, defined as at least three mitochondria in contact with each other (p=0.140539, young: 31.67 ± 1.67, n=6 mice, 60 boutons, aged Spd-: 19.07 ± 5.39, n=6, 67 boutons, aged Spd+: 27.78 ± 3.62, n=6 mice, 64 boutons, Kruskal-Wallis test).Values represent the mean ± SEM. Graphs show medians, interquartile ranges and min/max values. Circles are outliers, triangles are extremes.

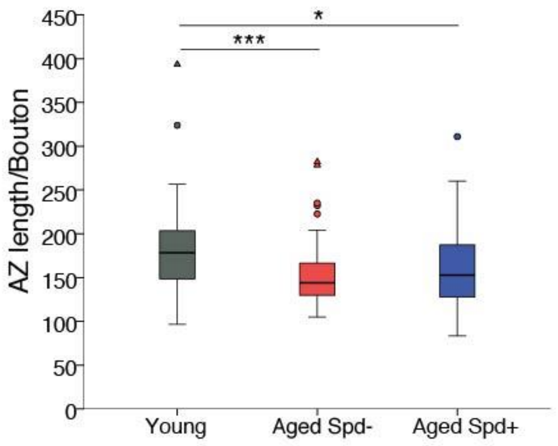

Fig. S2. Age-dependent decrease in mossy fiber active zone length is not rescued by spermidine treatment. Ultrastructural analysis of active zone (AZ) length at Mossy Fiber (MF) terminals in young (5 months), aged controls (Spd-, 24 months) and aged spermidine (Spd+) treated mice (18 months + 6 months treatment). Aging results in a significant decrease in AZ length ($p=0.000075$, aged Spd-: 154.36 ± 4.35, $n=67$ boutons; young: 182.83 ± 6.54, $n=60$ boutons, 6 mice per group, Kruskal-Wallis test followed by Mann Whitney U test with Bonferroni correction with α set to 0.016667). Six months long Spd treatment does not rescue the age-dependent decrease in AZ length (aged Spd+: 161.43 ± 5.44, $n=64$ boutons; aged Spd+ vs aged Spd-:$p=0.441929$; aged Spd+ vs young: $p=0.007039$, Kruskal-Wallis test followed by Mann Whitney U test with Bonferroni correction with α set to 0.016667). *$p<0.05$, ***$p<0.001$. Values represent the mean ± SEM. Graphs show medians, interquartile ranges and min/max values. Circles are outliers, triangles are extremes.

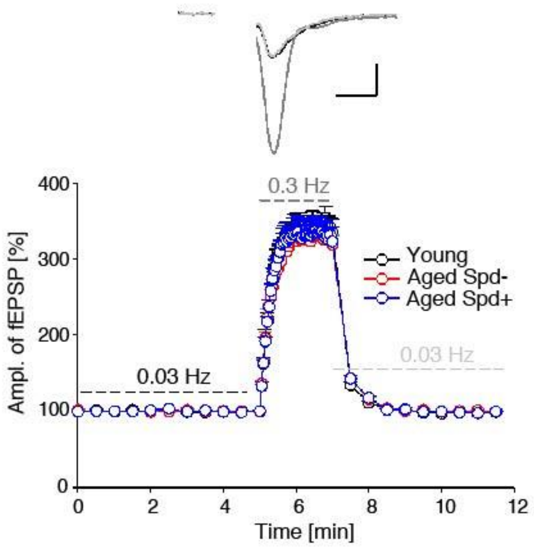

Fig. S3: Normal MF-CA3 synaptic frequency facilitation in aged control and aged spermidine treated mice. Frequency facilitation of MF-CA3 synapses at 0.3 Hz is not altered in aged control and aged spermidine treated mice. Characteristic profile of MF-CA3 synaptic facilitation following stimulation frequency switch from 0.03 Hz to 0.3 Hz showing normal frequency facilitation in aged and aged spermidine treated mice. Inset shows average of MF-fEPSPs collected before (black), after (grey) and at the end of frequency facilitation (dark grey).The numbers of tested slices n and mice N for each groups are respectively: young (5 months old) n=12 N=6, aged Spd- (24 months old) n=13 N=7, aged Spd+ (18+6months) n=11 N=6. Calibration: 0.5 mV and 5 ms.
